# A road map through the multi‐faceted world of female genital cosmetic surgical techniques

**DOI:** 10.1002/ijgo.16169

**Published:** 2025-01-19

**Authors:** Giovanni Buzzaccarini, Rebecca Susanna Degliuomini, Laura De Rosa, Silvia Villa, Silvia Messina, Gabriele Siesto, Massimo Candiani, Stefano Salvatore

**Affiliations:** ^1^ Obstetrics and Gynecology Unit, IRCCS San Raffaele Scientific Institute Vita‐Salute San Raffaele University Milan Italy

**Keywords:** cosmetic surgery, evidence‐based training, labiaplasty, perineoplasty, vaginoplasty

## Abstract

The demand for female genital cosmetic surgery (FGCS) has significantly increased in recent years for two reasons: the advancement of surgical operations and the increased attention to women's esthetic and sexual well‐being. Three authors independently analyzed different databases up to April 1, 2024. They selected the relevant data according to inclusion and exclusion criteria. Two team members retrieved and evaluated the full text of the articles for eligibility, extracted the data independently, and included populations, intervention type, and outcomes using a pre‐piloted standard method. Any disagreement over the eligibility of some articles was resolved through discussion with an external collaborator. Considering the growing interest in FGCS procedures, the present review aims to analyze the most up‐to‐date surgical techniques, to provide adequate evidence‐based surgical training, in order to avoid complications. Labiaplasty aims to reduce excess labial tissue. To perform this procedure, it is possible to implement several techniques, that have recently been updated to ensure the lowest rate of complications and the best esthetic result. Vaginoplasty involves strengthening the vaginal wall and repairing vaginal lesions or asymmetries. Perineoplasty, the technique of choice in all scenarios in which perineal trauma occurs, is achieved by strengthening the perineal body and restoring the rectovaginal fascia. Other procedures described are labia majora augmentation, mons pubis surgery, clitoral hood reduction, lipofilling, and hymen reconstruction. Our review emphasizes that robust, evidence‐based training is required before performing such procedures, to avoid ineffective and potentially harmful surgical practices.

## Synopsis

This study highlights, in a narrative method, the most significant approaches in female cosmetic gynecologic surgery.

## 1 INTRODUCTION

Esthetic genital surgical treatments, also known as Female Genital Cosmetic Surgery (FGCS), are defined as any vaginal or labial surgical modification of anatomy that is offered for esthetic reasons or to treat normal changes occurring throughout the lifespan. The growing interest in such procedures is due to the advancement of gynecologic esthetic surgical operations and the revelation of the need for more esthetic and sexual well‐being in women, following the previous demand for esthetic breast surgery .^1^ Conversely, different approaches can be identified for both vaginal and vulvar surgery with the aim of enhancing both esthetic appearance and functional outcomes. In particular, labia minora hypertrophy, which is in the spotlight for FGCS, can affect women's sexual well‐being in two main ways beyond the esthetic purpose.^2^ First, in young and fertile women, labia minora hypertrophy can impair sexual intercourse, especially during initial sexual encounters, due to psychological distress mainly caused by the widespread distribution of images and videos from the pornographic industry. The dissemination of such images can provoke in young women a sense of comparison, potentially leading to distress. Second, significant hypertrophy can hinder comfort during sexual intercourse because of the physical obstruction it causes. Third, in mature women, especially postmenopausal women, labia minora hypertrophy can lead to bruising and bleeding after sexual intercourse.^3^

The increasing spread of such procedures strongly advocates for proper evidence‐based provision, in order to avoid complications and post‐surgical chronic pain, which could affect women's sexual well‐being. In light of the increasing interest in FGCS procedures, we have produced an updated scoping review of techniques for both esthetic surgeons and cosmetic urogynecologists.

## 2 MATERIALS AND METHODS

The search was conducted by different researchers independently, evaluating several independent databases (MEDLINE, EMBASE, Global Health, Cochrane Database of Systematic Reviews, Cochrane Central Register of Controlled Trials, Cochrane Methodology Register) to find all possible relevant trials. The keywords used to find relevant articles were: “cosmetic gynecology”, “labia minora hypertrophy”, “vaginoplasty”, “labiaplasty”, “female esthetic surgery”, “female genital surgery”. Key criteria for inclusion were: (1) full articles in the English language and (2) original studies concerning female genital cosmetic procedures. All articles were screened using the keywords by three independent authors (GB, RSD, LDR). The search was conducted without date restriction until April 1, 2024.

The full text of these potentially eligible articles was retrieved and assessed for eligibility by two independent review team members (GB and RSD). Any disagreement between them over the eligibility of some articles was resolved through discussion with an external collaborator (SS). All the studies screened through the inclusion criteria were examined, and relevant data were extracted for each paper. Two authors (GB and LDR) independently extracted data from articles about study characteristics and included populations, type of intervention and outcomes, using a pre‐piloted standard form to ensure consistency. Due to the nature of the findings, we opted for a narrative scoping synthesis of the results from selected articles.

## 3 RESULTS AND DISCUSSION

### 3.1 Labiaplasty

Following liposuction, breast augmentation, and rhinoplasty, labiaplasty was reported to be the fourth most common cosmetic surgical procedure in the USA in 2013. The etiology of labia minora hypertrophy is not known, but several hypotheses have been proposed. It has been proposed that labia minora hypertrophy is a hormone‐related lesion, similar to fibroepithelial stromal polyps,^4^ or a manifestation of chronic lymphedema, rather than an anatomic variant.^5^ Initial hypotheses suggesting that labia minora hypertrophy was associated with masturbation or multiple pregnancies have been discarded.^6^ The primary indications for labiaplasty are (1) hypertrophy of labia minora with esthetic or functional impairment and (2) labial asymmetry.^7^

The main cause of labial hypertrophy is congenital. However, there are several acquired causes, such as exogenous androgen hormones in childhood, topical estrogen sensitivity, dermatitis secondary to urinary incontinence, vulvar lymphedema, and myelodysplastic diseases.^2^ Functional problems related to labia minora hypertrophy include pain while wearing tight clothes or during athletic activities. Moreover, psychological distress can result from the undesirable esthetic appearance. For this reason, the most common problem that labiaplasty deal with is dissatisfaction with labial appearance. Other common concerns are discomfort or pain during sexual activity, general irritation, and issues related to hygiene or infection. The primary goal of the operation is to obtain minimal or no protrusion of the labia minora beyond the labia majora when the patient is standing. To achieve this esthetic result, the labia minora should be symmetrical and hidden by the labia majora. There is a standard of the normal anatomy of the labia minora and, to some extent, the labia majora, but this is adaptable to individual differences. To avoid confusion in diagnosis, a simplified classification system has been created for labial protrusion based on the distance of the lateral edge of the labia minora from that of the labia majora. The labial protrusion is classified by Franco and Franco^8^ as class I (0–2 cm), class II (2–4 cm), class III (4–6 cm), and class IV (>6 cm). When approaching a labiaplasty, the main aim should include the size reduction of hypertrophic labia minora with the accurate maintenance of the neurovascular supply, preservation of the normal introitus shape, appropriate color/tissue matching, and minimal risk of complications.^2^ The procedure involves eliminating unwanted labial minora tissue, often bilaterally, although sometimes unilaterally in cases of natural asymmetry. It is also important to highlight the patient satisfaction rate for each labiaplasty technique. In the systematic review conducted by Motakef et al.,^2^ in 2015, the patient satisfaction rate was around 94%–100%.

Although there are many different techniques for labiaplasty, few studies defined an algorithm to match the degree of deformity with the optimal surgical procedure. According to Ellsworth et al.,^9^ patients with type I and type II labia hypertrophy can be treated more effectively with the de‐epithelialization technique, unlike patients with type III or IV labia hypertrophy, because of the inability in these patients to completely reduce labial volume and the poor esthetic results. These patients may be more suitable candidates for both the direct excision technique and the wedge resection technique.^2^ Ellsworth et al.^9^ also demonstrated that around 92% of the patients they enlisted in their studies were “very satisfied”.^9^ However, Crouch et al.,^10^ in 2011, suggested that labiaplasty should only be offered to women who have labia minora of more than 5 cm or an asymmetry greater than 3 cm. In 2015, Gonzalez et al.^11^ added two additional dimensions to Franco's classification in 2015: position of hypertrophy (anterior [A], central [B], or generalized [C] and symmetric [S] and asymmetric [AS]), which give a more complete description than previous classification systems. This classification system can make communication between doctors easier.^12^ Qiang et al.,^3^ in 2023, conducted a study on the reasons why Chinese women require labiaplasty: compared with the West, this technique is used in China, mainly for functional reasons.^3^

There is a cultural difference between East and West. Eastern women are more conservative in their characteristics than Western women and less willing to talk about private topics like this. However, from the questionnaires administered in this study, it was found that they tended to have a negative genital self‐image and, to some extent, a lack of confidence. In addition, an important finding reported that 63.9% of participants have a sexual partner and 6.9% were influenced by their male spouse. The esthetic aspect goes hand‐in‐hand with the functional one: having a positive body image of oneself is related to a higher level of sexual satisfaction.^3^ Another factor that affects the esthetic perception is represented by the media: public images of female genitals make women reflect on their appearance, leading women to believe that surgery is necessary to obtain the ideal appearance.

It is believed that labiaplasty can affect the psychological well‐being of adolescent women. The truth is that social and cultural pressures produce esthetic standards that can negatively affect self‐esteem and cause shame. As a result, there is a conflict between the autonomy in wanting to change your genital appearance to conform to these ideals, and the awareness that such a change could alter the natural body.^12^ For vulvar surgery to be justified, it is essential that it is performed within a strict regulatory framework that protects the patient's autonomy and guarantees the principle of nonmaleficence.^12^ Current guidelines state that the patient's motivations must be investigated.^12^ All of these aspects must be fully understood by the patient and clarified in the informed consent that will be signed before the procedure, ensuring that every decision is made in a conscious and responsible way.^12^ To approach young girls who require labiaplasty, the gynecologist should have an in‐depth knowledge of all the non‐surgical possibilities that improve appearance and function, perform a detailed consultation that takes into account the motivations of expectations, recognize dysmorphophobia, assess the patient's maturity and autonomy, and explain complications. This speech makes us reflect on how much the patient can be influenced by social pressures: adolescence is the period in which one's sexual identity develops and probably many girls have an idea of genital normality distorted by social and cultural expectations. One approach to improving anatomical and functional knowledge of one's genitalia could be through education in schools. This could help to boost self‐esteem and genital appreciation, leading to a healthier body image, a better quality of life, and possibly a reduction in the desire for genital surgery. Today, young people have easy access to a wealth of information through the internet and social media, but finding reliable sources can be challenging, especially due to the prevalence of digitally altered images of genitalia.

**FIGURE 1:**
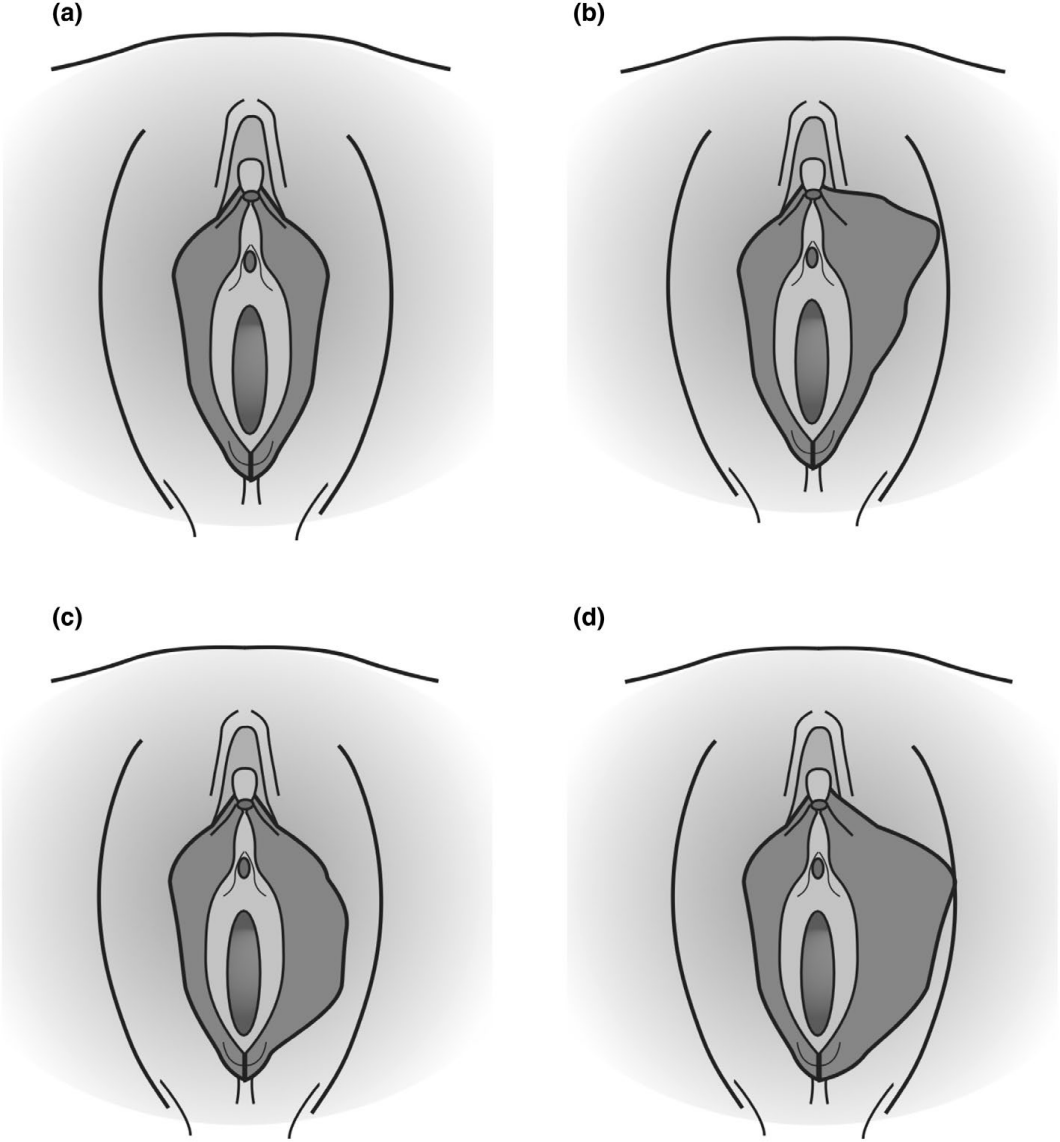
(a) Normal female genitalia. (b–d) Classification of labia minora hypertrophy proposed by Franco and updated by Gonzalez in 2015, based on the position: (b) anterior hypertrophy; (c) central hypertrophy; and (d) generalized hypertrophy.

There are various techniques for labiaplasty and they are described below (Figure 1).

#### Edge resection technique

This was the first labiaplasty technique described in the literature. It involves creating a linear vertical incision on the labia minora and removing excess labial tissue by means of an excision line that follows the curve of the labia. The long suture line in the vertical direction causes loss of the natural contour, staining, and texture of the free edge of the labia minora and it can lead to visible scarring due to chronic irritation because of contact with clothing.^2^, ^12^ Different techniques have emerged in the literature to prevent labia scar retraction, chronic irritation, and clenching of the introitus:

- Maas and Hage,^13^ in 2000, developed a W‐shaped resection, an interdigitated suture of the protuberant labia minora often used in plastic surgery. The closure of the opposing W‐shaped incisions results in a tension‐free zigzag suture line that runs obliquely across the edge of the labia.^14^ This technique has the following advantages: wound contraction (both longitudinally and transversely), reduced risk of wound dehiscence, and posterior fourchette advancement. This technique leaves the anterior and posterior commissure and the tissue around the base of the labia minora intact. It does not affect the branches of the superficial entrance of the perineal nerve, thus preserving sexual function and sensation.^13^
- Two other techniques used with the aim of avoiding scar contraction are the S‐shaped resection introduced by Felicio et al. and the Z‐plasty, widely used in plastic surgery. Straight resection can be performed with a scalpel, diathermy, or a combination of both. Some surgeons use a clamp to squeeze the incision site first and to reduce blood loss.^12^ Complications are usually related to excess tissue resection, which can result in complete amputation of the labia minora or a centrally shortened labia minora.

**FIGURE 2:**
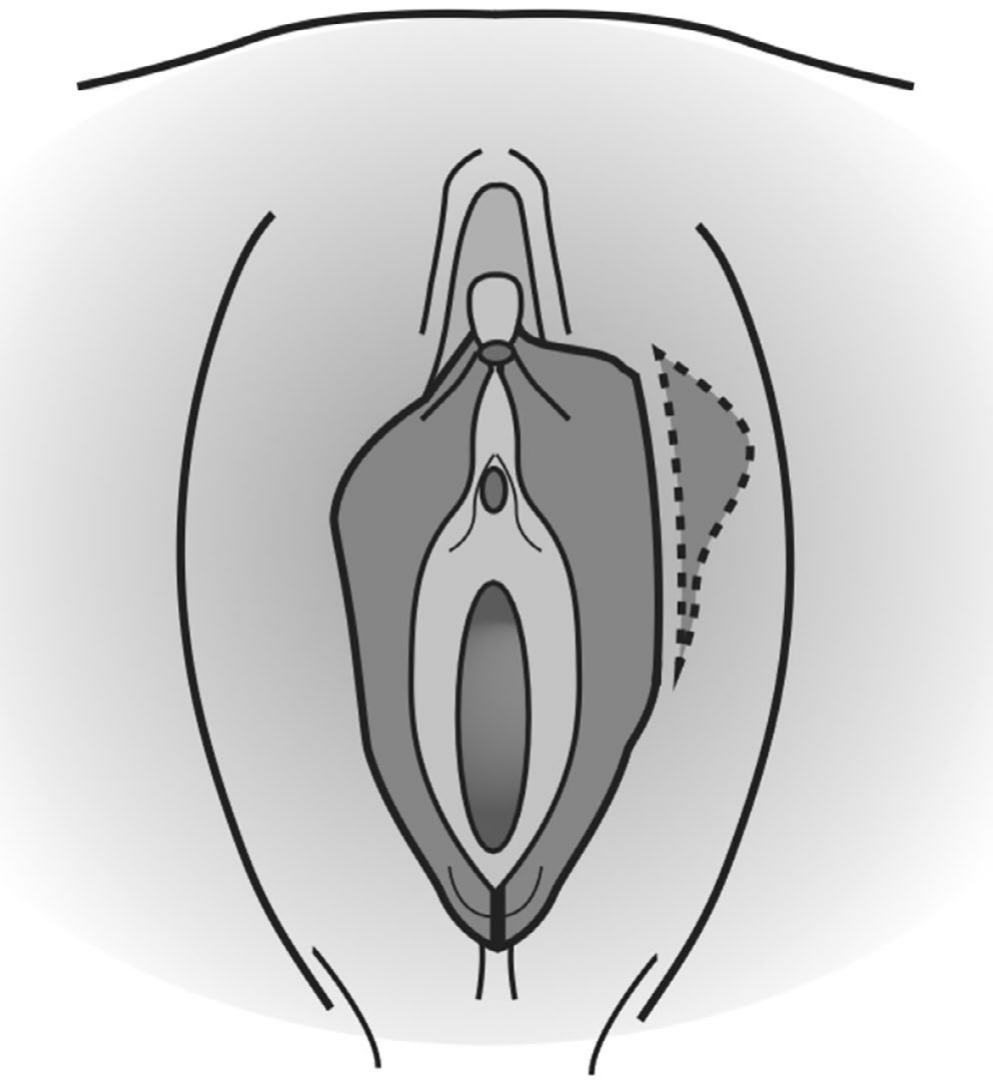
Edge resection technique: Consists of creating a linear vertical incision on the labia minora and removing excess labial tissue by means of an excision line that follows the curve of the labia.

For patients with a prominent clitoral hood before surgery, persistent protrusion and increased volume of the clitoral hood can occur as a potential complication.^15^ Clitoral hypertrophy is corrected with a fusiform excision lateral to the clitoris on each side, so that the prominent part is exposed. A complication of correcting both labial and clitoral surgeries is a more prolonged edema, possibly persistent for up to 3 months. Wound repair is performed with continuous absorbable stitches of 4–0 Vicryl or 4–0 Chromic Gut.^16^ Lange et al.^17^ updated the indications and limitations of the edge resection or trimming technique in 2023. They introduced an algorithm that leads to a choice of the correct technique through five decisional steps (Figure 2).

#### Wedge resection technique

**FIGURE 3:**
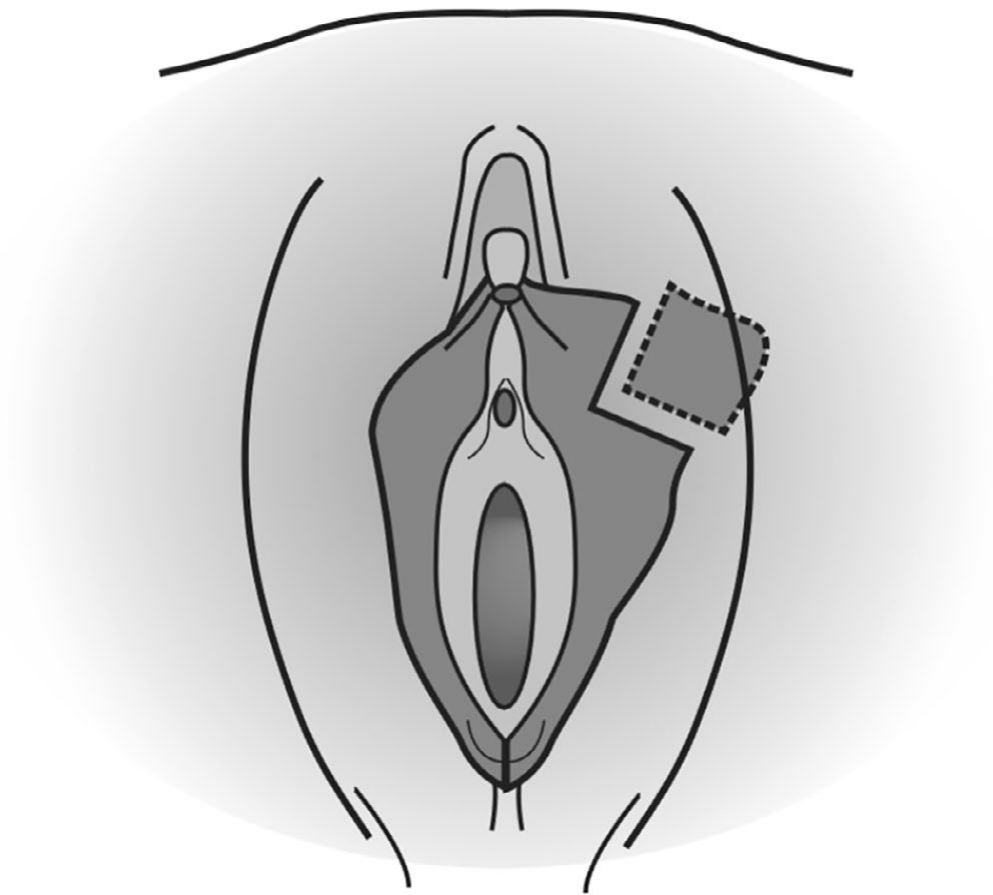
Wedge resection technique: After drawing the limits of resections, the edges are cut with a scalpel and then approximated. This technique allows for a noticeable reduction of the labia while maintaining a natural edge.

**FIGURE 4:**
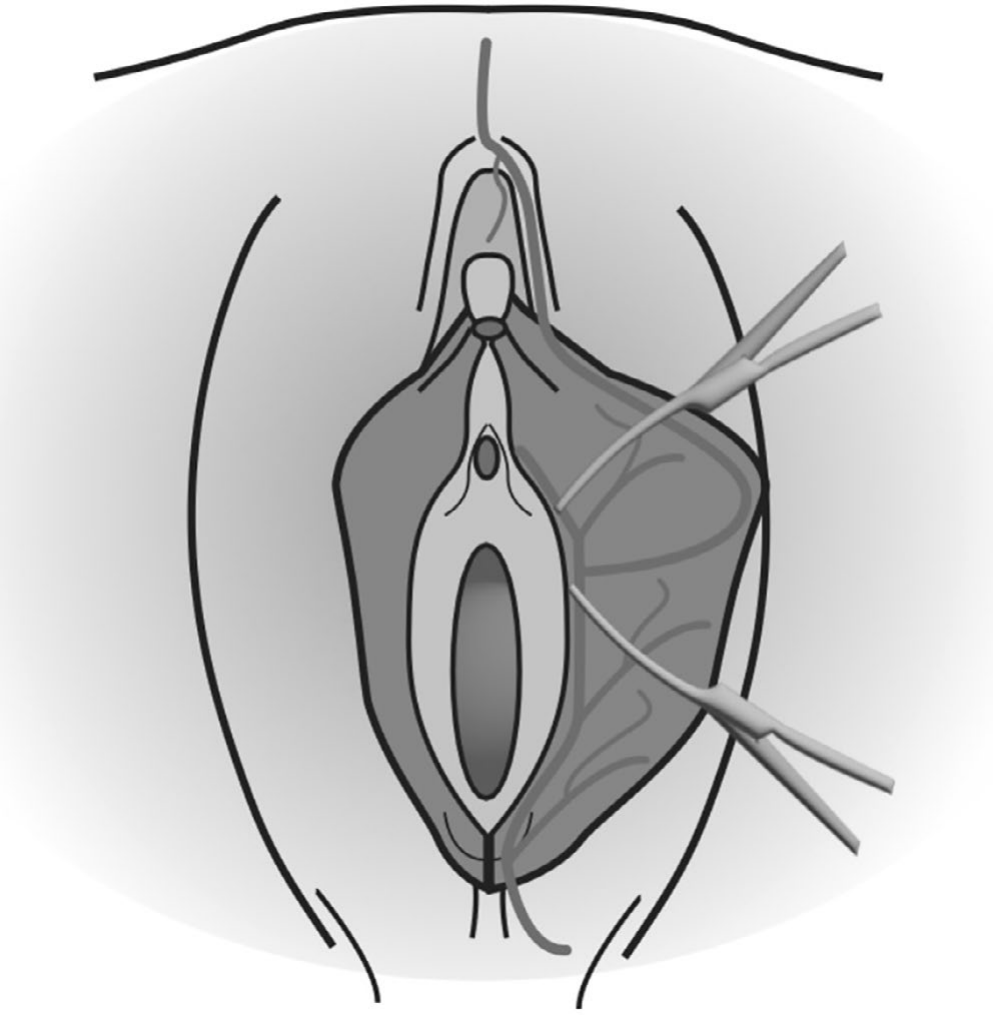
To perform wedge resection, two clamps are placed around the area to be cut: The first clamp is placed on the posterior part with an angle of 30° to the base, without impeding the blood supply, and the second clamp is positioned away from the clitoris.

Its main indication is protrusion of the labia minora extending at least 2 cm beyond the fourchette (Figure 3). It is the most popular labiaplasty technique and has been developed to improve esthetic results, as it preserves the shape and color of the labia, or to prevent loss of function or sensation. The labial artery can be identified and preserved before execution, but the technique is often performed without this step.^12^ It is performed under general anesthesia. First, it is important to draw the limits of resections and to place two Kochers. The first clamp is placed on the posterior part with an angle of 30° to the base, without impeding the blood supply, and the second is positioned away from the clitoris^18^ (Figure 4). The edges are then cut with a scalpel and then approximated, without using coagulation techniques and leaving an excellent esthetic suture without free scars on the edge: Vicryl 4.0 is used for the suture on the labia and Vicryl 4.0 or 5.0 or Monocryl 5.0 for the skin.^12^ This technique allows for a noticeable reduction of the labia while maintaining a natural edge.^19^ There is a high risk of wound healing complications because of tension at the edge of the repair; most surgeons report central or edge dehiscence, flap necrosis, discomfort, or superficial infections.^2^, ^12^ However, it reduces the risk of significant clitoral cap sagging after labiaplasty compared with the edge resection technique. Wedge resection maintains the natural contour and coloring of the free edge of the labia minora, but can create an abrupt contrast in the coloration of the labia minora where the tissues are reapproximated. The labia cannot be reduced too much, and this is an advantage, unlike the rim resection technique, in which care must be taken to leave sufficient labial length so that the patient does not experience dryness, infection, vaginal discharge, or lengthening of the vaginal introitus during sexual intercourse.^2^, ^12^ More recent studies, such as those conducted by Abbed et al.^20^ and by Hamoud et al.,^18^ in 2018 and 2020, respectively, suggest that the wedge resection technique is the favorite among surgeons. However, it is still little used by gynecologists.

#### Central wedge resection technique

This technique is carried out by drawing two Z‐shaped incisions at 90° on the inner and outer surfaces of both lesser labia, using predefined patterns. These two Z‐shaped incisions converge from the free edge of the central third of the labia to a common origin at the base of each labium, in the direction of the ventral part of the introit. The labial wedge located between both Z‐plastics is removed by 90°, with careful hemostasis of the fine vessels. Finally, the edges of the resection are directly sutured with single stitches with Vicryl 4–0 suture.^21^ To also perform the reduction of the clitoral hood, the central wedge technique is combined with an anterolateral curved excision of the redundant lateral labia and the excess of the lateral part of the clitoris.^12^ In 2020, Filho et al.^22^ described a technique to facilitate the execution of the central wedge resection technique: the labia minora are sutured on the inside of the thigh by configuring a butterfly open‐wing design.^22^ Qiang et al.,^23^ in 2021, conceived a new concept for central wedge resection during labiaplasty: the technique involved leaving two non‐parallel closure lines on the lateral and medial surfaces; so, the incisions were on different levels. It is performed by tilting the edge of the scalpel during the skin incision, to preserve more subcutaneous tissue. Then, the first incision was made with the scissors along the bottom line of the wedge upwards and inwards, and the second one was made in a downward and inward direction along the top line of the wedge.^23^ The advantage is to avoid wound dehiscence and scar contracture, but the downside is that it causes color changes on the labial edges. It is not recommended for dark‐skinned patients.

#### Extended central wedge resection technique

This consists of a wider resection of the labia minora.^24^ This technique arises from the need to avoid creating a narrow intake and/or a separation of the suture line, excess redundant clitoral tissue, and postoperative pain. The protuberant part of the labia minor is removed with a central or V wedge and the wedge is cut to avoid tension on the suture line. In order for the intake to not be pathologically narrowed, there must be no tension, except if there is a high posterior vaginal labium. In this case, you may need to release it. The clitoral frenulum, which extends to the upper labrum, is used as a guide for aligning the upper labium to the lower one in marking the wedge excision. The internal excision has a V‐shaped extension into the vagina. The excision of the outer wedge is lateral and anteriorly curved to remove the redundant lateral labium and excess clitoris, only if the patient wishes. For this reason, to ensure that subcutaneous tissue is preserved, and the edges are brought closer together, the internal and external V‐shaped excisions have different shapes. In some cases, the clitoris skin may protrude more medially along the hood. In such instances, the lateral incision is made only as far as the lateral labium, and a vertical elliptical incision is made from the clitoral hood to remove this excess skin. It is essential to close the subcutaneous tissue correctly to avoid fistulas: two or three layers are made according to the thickness of the lip with a 5–0 Monocryl suture.^19^

#### Inferior wedge resection technique

In this case the wedge is performed more inferiorly, and it is preferred when the labia minora are wider inferiorly. This technique helps in reaching anatomic results that are satisfactory for 93% of patients; the other 7% undergo secondary labiaplasty because of minimal wound dehiscence that resulted in an imperfect esthetic outcome.^25^ One clamp is placed on the posterior part of the labia, and another is positioned to form a 90° angle with the first. The V‐shaped area of redundant labial tissue bounded by the two clamps is excised, leaving an anterior flap that will form a minor labium of the desired size. The anterior labial flap is gently stretched out, placed without tension, and secured to the underlying connective tissue with a 4–0 Polyglactin suture. Continuous sutures are used to close the surface under the flap and the inner and outer edges of the flap to the vulvar mucosa.^25^ An improvement of the original technique described by Alter^19^ in 1998 and modified by Rouzier et al.^25^ in 2000 was proposed by Munhoz et al.^26^ in 2006: in their studies they described the procedure based on the resection of the lower wedge of the labia minora and the reconstruction of the upper pedicle flap. The technique is indicated in patients with moderate to large labia minora hypertrophy with a size greater than 3 cm (measured horizontally from the midline applying minimal lateral tension). For this reason, a thorough inspection of the genitals before the operation is indicated. The pinching test is also performed: a small forceps is placed on the central part of the labia minora (point A) which is stretched inferiorly, towards the back of the vagina (point B). In case of skin tension, the forceps are moved upwards; Otherwise, if skin laxity is noticed, the forceps are moved down to resect more tissue. A wedge‐shaped area located between the two points, the edge of the labia and its base, represents the area of tissue to be resected, which varies depending on excess tissue and skin‐mucosal laxity. According to this procedure, it is possible to simulate the final esthetic result and estimate the amount of tissue that needs to be resected and the extension of the upper flap. The upper wedge‐shaped flap is designed on the remaining top of the labia minora between the A‐spot (tip) and the clitoral region (base). For moderate hypertrophy, the area to be resected is planned as an isosceles triangle located at the bottom of the labia minora. In cases of significant hypertrophy, the tissue to be excised may extend to the anterior region of the labia minora. The incision lines are then drawn more obliquely with curved edges, creating a convex shape. This approach allows for the removal of a larger portion of skin and mucosa, resulting in a thin upper flap.^26^ First, local anesthesia is performed: this promotes vasoconstriction and separation of the layers, increasing the virtual space of the subcutaneous tissue, to favor the technique. A cutaneous incision is made in the subcutaneous tissue on the medial layer of the labium minora and a complementary one on the lateral layer by resecting the total area in a wedge shape and suturing with absorbable stitches.^26^

#### Posterior wedge resection technique

This was described in 1971 by Martincik and Malinovsky^27^ and is also known as “resectio cuneiformis labii minoris”. It is another example of the progression and refinement of the technique that occurs as an increasing number of patients require these procedures. Similar to the inferior wedge resection described by Rouzier et al.^25^ in 2000 and later by Munhoz et al.^26^ in 2006, an upper‐base peduncle is used. However, most excision is posterior to the outer labium, between the outer and inner labia minora, and it retains the outermost or anterior edge of the labia minora, especially at the upper and lower boundaries.^28^ The line of incision is performed starting from the lateral border of the labia minora (to preserve the natural pigment and tissue) and continuing infero‐superiorly and proceeding medially down to the base of the labia minora, approximately 1 cm up to the frenulum. The line then continues posteriorly, to the posterior fourchette and is stopped before reaching the midline.^28^ About 1 cm of the posterior fourchette has been left intact, to create a more natural look. A “tailor tack” approach was employed to trim any excess labial tissue and the flap was sutured in three layers, all with interrupted Vicryl 4–0 stitches, placed along the natural folds to ensure minimum visibility. If there is asymmetry between the left and right labia minora, resection is performed on the side with greater hypertrophy with sharp scissors to cut a full‐thickness flap. These steps helped to achieve symmetry and achieve good results with easy reproducibility. In 2013 Kelishadi et al.^28^ described two complications: a mild wound dehiscence caused by early sexual intercourse at 2 weeks that healed by secondary intention and a hematoma immediately after the operation, evacuated without further sequelae. This technique is especially indicated when dealing with extreme degrees of hypertrophy of the labia minora, when the darker and wavier edge of the labia minora has an aged appearance, and when the outer labial edge is thicker than the most proximal portion.^28^

#### De‐epithelialization technique

This technique was introduced by Choi and Kim^29^ in 2000 and consists in de‐epithelialization (creating an ellipsoid incision on the inner side of the labia minora) of the central portion and re‐approximation; it reduces the length of the labia minora and minimizes the thickness. There is a higher risk of neurovascular damage and complications such as hematoma formation with this procedure. Choi and Kim^29^ created a triangle shape in the center of the labia minora and, after injection of lidocaine and adrenaline, the central part was de‐epithelialized with a scalpel. Ostrzenski,^30^ on the other hand, in 2014, marked the amount of tissue to be removed centrally in the labia minora. The inner and outer surfaces of the labia minora are sutured separately without suturing the erectile tissue between them.^12^ This preserves the natural color, contour, and texture of the edge of the lesser lips. This labiaplasty is performed by deepening the middle part of the labia minora and reapproximating the surfaces. Because full‐thickness tissue is not cut, this technique is most useful for very mild hypertrophy. The technique has the limitation of not being able to drastically reduce labial volume in patients with larger labia minora and, when the edges are brought together, results in an inappropriately large labial base and a poor esthetic outcome.^28^ Cao et al.^31^ further modified this technique in 2012, resulting in de‐epithelialization of the posterior part of the labia minora. In 2013, Mayer^32^ highlighted that a complication of this technique may be the excessive thickness of the labial base, due to the vertical “telescopation” of the subcutaneous tissue.^33^

#### Composite reduction labiaplasty

This procedure combines labial tissue reduction and removal of tissue located cranial or caudal to the clitoris.^34^ This technique divides the tissue into two distinct sections, arranged to allow for an even reduction of the labia along their full length, with particular focus on the clitoral hood area. It also provides the possibility of addressing a protruding clitoral glans (clitoral protrusion) if necessary. An improvement of sexual excitability is seen in approximately 35% of patients,^34^ especially among those who underwent correction of clitoral protrusion. This can be related to the fact that with the clitoris positioned closer to the vaginal introitus, it is exposed to more direct stimulation. A radiofrequency surgical device is used for the skin incision, which is more precise and avoids tension. A curved line of incision is made caudally along the inner part of the labium minor towards the clitoris until it reaches the frenulum. It is important to preserve a triangular flap that represents the clitoris‐labial triangle, which is a fundamental structure of support and sensitivity in sexual intercourse. The incision line then turns caudally again and cuts a flap about 2–3 cm long with a rather narrow base and preserving enough subcutaneous tissue to prevent bleeding. From here the incision line continues in parallel to the crease of the labia majora and then takes on an S‐shape to run along the outer part of the labium, returning to the start. The excess tissue can then be removed and, subsequently, a crescent moon under the clitoris and a rectangle above the clitoris is also cut, which is then closed with continuous Vicryl 5–0 sutures because monofilament sutures can cause painful postoperative irritation of the clitoris (in fact, they are used for the main closure). This technique reduces the length of the labia and narrows the hood of the clitoris, which moves inside the labia majora.^34^ Composite reduction is a method that effectively yields excellent esthetic results. However, it is also associated with the highest reported complication and reoperation rates in the literature, at 17,4%.^2^

#### Custom flask labiaplasty

This technique permits precise reduction of the labia minora in the necessary regions, in a customized manner, to achieve symmetry and a natural appearance of skin and to maintain the neurovascular pedicles. The main advantages of this technique are preservation of the neurovascular supply of the labia, predictability and reproducibility of results, and a low rate of complications.^11^ The labia minora are supplied by small arterial branches that originate from the anastomosis of the external superficial pudendal artery (femoral artery branch) and the internal pudendal artery (internal iliac artery branch). The labia minora are innervated by the posterior labial nerve, which is a continuation of the pudendal nerve, which also branches into the dorsal nerve of the clitoris.^35^

#### Fenestration labiaplasty technique

This involves an inferior flap transposition, which obtains reductions in both height and length of the labia minora. The labium is separated into two segments: the upper portion, which is partially separated from the rest of the labium, and the lower section, located at the base of the labia minora. Then, symmetry is established and the natural color and contour of the labium tends to be preserved. A natural appearance of the labium frenulum (posterior edge of the fossa navicularis) must be also considered.^30^

Labiaplasty may cause disfigurement, scarring, loss of sensation or hypersensitivity, dyspareunia, infection, dissatisfaction, or repair separation. Clitoral hood surgery can also lead to scarring with hypersensitivity and damage to the hood. There are also risks of infection, hematomas, and abscess formation.^33^, ^36^

### 3.2 Vaginoplasty

Vaginoplasty encompasses procedures for vaginal tightening and the rectification of vaginal lesions or deformities. These procedures can be performed under loco‐regional anesthesia, with complication rates being low (2%–4%) and including: dyspareunia, reduced lubrication, constipation, wound infection, hemorrhage, suture failure (predominantly in the perineum), buttock pain, and perforation of the rectal mucosa. The significance of classification has been highlighted by Ostrzenski.^30^ He devised a classification for a wide vagina from category A to D, based on the presence or absence of columnar rugae and site‐specific defects.^30^ These clinical factors are vital for diagnosing and choosing the correct surgical approach. Women may undergo this surgery for various reasons, including purely cosmetic, functional impairments like pain and discomfort, or both. The psychological impact of genital appearance, notably the labia minora, leading to substantial emotional distress, especially among adolescents, should not be overlooked.^2^ Typically, the procedure involves tightening the anterior vaginal wall and folding the vesicovaginal fascia, though some surgeons opt to tighten the lateral wall to circumvent a posterior scar, where there is increased pressure and sensitivity. The posterior vaginal wall is tightened by folding the rectovaginal fascia and bringing the levators closer together. Risks include bleeding, hematoma, injury to the bowel or bladder, rectovaginal fistula scarring, vaginal stenosis, dyspareunia, and changed sensation.^37^

### 3.3 Perineoplasty

Perineoplasty is indicated for esthetic reasons, a loose vaginal introitus, dyspareunia or diminished sexual satisfaction, or in the case of prolapse to provide third‐level support according to the DeLancey classification.^37^ Perineoplasty is the technique of choice in all scenarios where perineal trauma occurs, which can impact the neurologic and vascular functions of the tissues, underpinning sexual dysfunctions. Childbirth is one of the causes of trauma, particularly lacerations or episiotomies during labor, and weakening of the pelvic floor muscles, leading to incontinence, prolapse, and sexual dysfunction.^38^ In the absence of definitive guidelines on perineoplasty's use, Ulubay et al.^39^ undertook various studies on this technique for treating vaginal laxity, attributed to insufficient perineal support. The objective was to restore the integrity of the rectovaginal fascia and perineal body through perineoplasty. Following anesthesia (general or local) and intraoperative prophylaxis with 2 g of cefazolin, Allis clamps are placed bilaterally at the hymen's edge on the posterior vaginal fork, and a transverse incision is made with a number 11 scalpel. A clamp is used to elevate the perineum, cutting vertically from the posterior fork's center to the anus with the same scalpel. The perineal body is reinforced and approximated to the rectovaginal fascia with interrupted sutures; the superficial transverse perineal muscle is approximated along the midline with transverse interrupted sutures, and the bulbocavernosus muscle at the level of the posterior fourchette. This study was the first not to employ vaginal mucosal excision in conjunction with perineoplasty, indicating that vaginal mucosal excision leads to excessive fibrosis, reduced blood flow to the vagina, and consequent vaginal dryness and dyspareunia.^39^ Austin et al.,^1^ in 2019, described a posterior vaginoplasty technique with perineoplasty to narrow the vaginal introitus. The terms are variably used, often in conjunction. Initially, triangular‐shaped surgical markings are made, an inner triangle along the posterior wall of the vaginal canal and an outer triangle on the perineum, meeting at the hymen's level (widest resection point) to create a diamond‐shaped incision. An Allis clamp placed at the inner triangle's tip aids in visualization and access to the posterior vaginal wall. Starting at the hymenal annulus, a short, superficial incision is made along the midline in the posterior vaginal mucosa with a number 15 scalpel. It is vital to stay within the submucosal plane during midline dissection to avoid harming the perirectal vastus venous plexus and the anterior rectum itself (an intraoperative rectal examination may be conducted if in doubt). Following midline mucosa division, the flaps are elevated laterally in the submucosal plane and amputated, facilitating direct re‐approximation and repair. In the submucosal layer, sutures are aligned parallel to the mucosal surface to prevent damage to the rectum and perirectal venous plexus. After re‐approximating the bulbocavernosus and superficial transverse perineal muscles, the introitus and vaginal canal diameter are significantly reduced.^1^ Perineoplasty has also proven effective in correcting sexual dysfunctions, as evidenced by Inan et al.,^38^ in 2015: rhythmic contractions of the perineal muscles (particularly the levator ani muscle) are crucial for female sexual satisfaction. In instances of muscle hypotonia, orgasm intensity may be diminished; approximating the superficial perineal muscles along the midline and fortifying the perineal body is beneficial.^38^ Following perineoplasty, approximately 90% of patients report enhanced satisfaction with sexual intercourse.^37^

### 3.4 Other FGCS procedures

Labia majora augmentation is a cosmetic procedure aimed at enhancing the size and shape of the labia majora, the outer folds of skin surrounding the vulva. This technique can involve the use of autologous fat, which is fat harvested from another area of the patient's body, or synthetic filler materials such as hyaluronic acid. The procedure is designed to increase volume, improve contours, and potentially enhance the overall esthetic appearance of the genital area due to hygroscopic properties of the hyaluronic acid.^37^

Mons pubis surgery, specifically focusing on reconstruction and reduction, is designed to address and remove excess fatty tissue in the mons pubis region and potentially the upper parts of the labia majora.^40^ This is typically achieved through liposuction, a process that carefully extracts unwanted fat, resulting in a more esthetically pleasing contour and reducing any prominence in the area.^37^

Clitoral hood reduction, often performed in conjunction with labioplasty, represents one of the most frequent reasons of reoperation if not performed during labiaplasty.^24^ Recent studies^41^ underline the importance of clitoral hood reduction, because if the redundant clitoral prepuce is not corrected at the same time as labiaplasty, the result may be poor esthetically. Li et al.^41^ preferred to proceed in steps and first reduce the clitoral hood and then correct the hypertrophy of the labia. This technique differs from traditional techniques, in which the correction of the clitoris occurrs simultaneously with the correction of the hypertrophy of the labia.

Lipofilling is a popular reconstructive technique that uses an extemporaneous, autologous fat material. Fat grafting using the Coleman technique^42^ is simple, safe, reliable, and time effective. Palpable fatty cysts are a potential complication of this procedure.

Hymen reconstruction is also known as hymenoplasty, repair/reconstruction of the hymen, or revirgination. This is a controversial procedure that attempts to repair the hymen to a “virginal” state. This is most often accomplished by suturing hymenal remnant portions back together and is often done in combination with vaginoplasty to simultaneously decrease the caliber of the vagina.

Some of these procedures are administered for non‐esthetic purposes. They can be used for persistent vulvar or labial irritation, genital pain, labial hypertrophy, repair of female genital cutting, asymmetric growth of the labia secondary to congenital conditions and hypertrophy in the setting of excessive androgen hormone exposure.^40^, ^43^

## 4 CONCLUSIONS

The proliferation of FGCS procedures underscores the need for certified academic training and robust, evidence‐based medical practices to preclude ineffective and potentially harmful surgeries, conducted by unqualified or inadequately trained esthetic surgeons and cosmetic urogynecologists. Our comprehensive review addresses this requirement by presenting current evidence on FGCS techniques. We emphatically support the necessity for thorough surgical training before the performance of such procedures.

A limitation of the current body of research is the lack of prospective clinical studies. Despite the significant increase in the popularity of this procedure, the available literature remains limited, and current practices are poorly described. There is still a lack of consistency in how these procedures are carried out.^2^ Future studies should aim to establish or recommend standardized practices to optimize patient management, provide standardized measures for outcomes, validate current practices, and define the optimal approach to patient care.^33^

## AUTHOR CONTRIBUTIONS

G.B., R.S.D., S.S., and L.D.R. contributed to ideation and wrote the first draft of the manuscript. S.S, M.C., G.S., S.M., and M.R.C. contributed to editing and data retrieval. GB, SS, and LDR wrote the final version of manuscript.

## CONFLICT OF INTEREST STATEMENT

The authors have no conflicts of interest.

## Data Availability

Data sharing is not applicable to this article as no new data were created or analyzed in this study.
